# Insights into the detection of AMPA cross-reactivity: comparing cyclic peptide- to protein-based assays

**DOI:** 10.1186/s13075-025-03591-y

**Published:** 2025-07-07

**Authors:** Roxane Biersteker, Aegli Athanasiadou, Stef van der Meulen, Tineke J. van Wesemael, Linda M. Slot, Theresa Kissel, René E. M. Toes, Leendert A. Trouw, Diane van der Woude

**Affiliations:** 1 Department of Rheumatology, Leiden University Medical Center, Albinusdreef 2, Leiden, 2333 ZA The Netherlands; 2 Department of Immunology, Leiden University Medical Center, Albinusdreef 2, Leiden, 2333 ZA The Netherlands

**Keywords:** Anti-modified protein antibodies, ACPA, Autoantibodies, Cross-reactivity, Rheumatoid arthritis, Antigen backbone, Post-translational modifications

## Abstract

**Background:**

Autoantibodies targeting antigens carrying distinct post-translational modifications (PTMs), including citrullinated, carbamylated, and acetylated residues, are characteristic for rheumatoid arthritis (RA). These anti-modified protein antibodies (AMPAs) are typically detected using enzyme-linked immunosorbent assays (ELISAs), with peptides or protein antigens carrying these modifications. AMPAs exhibit significant cross-reactivity towards multiple PTMs, and increased cross-reactivity before disease onset may serve as a biomarker of disease progression. However, the impact of antigen backbone variations on cross-reactivity detection remains unclear. Therefore, we investigated how PTM-backbone variations affect AMPA-reactivity detection.

**Methods:**

Sera of 608 RA patients from the Early Arthritis Clinic (EAC) were measured for AMPA reactivity using modified fetal calf serum (FCS)- and cyclic peptide (CXP2)-based ELISAs. To investigate cross-reactivity patterns, we isolated AMPAs from serum using either modified FCS or peptides and assessed the reactivity of the isolated antibodies towards three different PTMs.

**Results:**

CXP2-based assays reveal a higher proportion of patients with serum reactivity against multiple PTM residues, while FCS-based assays exhibit a more restricted serological profile. When comparing responses to citrullinated versus carbamylated backbones, 61.2% of samples reacted to both PTM-residues on CXP2, while on FCS this percentage significantly decreased to 54.0%. The antigen backbone also influences AMPA isolation, as modified FCS captures AMPAs with a more restricted, less cross-reactive epitope recognition profile compared to those captured with modified peptides.

**Conclusion:**

Antigen backbones influence the detection of AMPA cross-reactivity. Gaining a better understanding of how PTM backbones affect this detection could provide insights into the structural basis of AMPA reactivity, and refine data interpretation by highlighting how assay choice influences results.

**Supplementary Information:**

The online version contains supplementary material available at 10.1186/s13075-025-03591-y.

## Introduction

Rheumatoid arthritis (RA) is a systemic autoimmune disease, marked by chronic joint inflammation and irreversible joint damage [1]. A hallmark of RA is the presence of anti-modified protein antibodies (AMPAs) in blood and synovial fluid [2, 3]. The first AMPAs identified were directed towards epitopes containing deiminated arginine (i.e. citrulline), known as anti-citrullinated protein antibodies (ACPAs). Subsequent research has identified antibodies targeting other post-translational modifications (PTMs), such as carbamylated or acetylated lysine, known as anti-carbamylated protein antibodies (anti-CarPs) and anti-acetylated protein antibodies (AAPAs), respectively [4, 5].

While ACPAs offer over 95% disease specificity for RA, other AMPAs, although present in RA, may also be detected in other rheumatic diseases [6, 7]. For this reason, only ACPAs are part of the ACR/EULAR RA classification criteria [8]. Apart from their diagnostic value, AMPAs may also actively contribute to the pathogenesis of RA since their presence is often associated with poorer prognosis and more severe joint damage [9, 10]. Consequently, AMPAs continue to be a major focus of research interest, and several different assays have been developed for their detection. In enzyme-linked immunosorbent assays (ELISAs), PTM proteins such as vimentin, enolase, fibrinogen, or fetal calf serum (FCS), and PTM peptides have been employed as antigenic substrates. In contrast to modified proteins, synthetic peptides offer precise control over the exact epitopes displayed and the specific locations of PTM-modified amino acids within their sequences. Currently, several generations of cyclic peptide-based assays are being utilised in clinical and experimental practice [11, 12].

Over the years, AMPAs have been studied extensively both at the polyclonal and monoclonal antibody level. It has become apparent that AMPAs not only display diverse fine-specificity profiles, reacting to either a broad or narrow array of PTM proteins and peptides, but also exhibit cross-reactive properties as they recognise multiple PTMs [13]. This inherent cross-reactivity has been observed in pre-disease stages and in ACPA-positive non-RA patients [14]. Since reactivity to multiple PTM epitopes, and thus possibly also cross-reactivity, has been found to increase in the period leading up to disease onset, developing tools to study these features is crucial for their potential as biomarkers of disease progression. Currently, the extensively optimised citrullinated peptides used in diagnostic laboratories have been designed to effectively distinguish RA patients from controls, likely facilitating the detection of multi-reactive antibodies [15]. It is key to exactly characterise the influence of PTM backbones on AMPA detection to better understand structural differences in AMPA cross-reactivity and improve diagnostic AMPA assays. As evidenced by monoclonal antibodies, AMPA reactivities towards different PTM backbones can vary, however, it is unknown to what extent these backbones can affect the degree of AMPA cross-reactivity detection in serum [13–16]. To investigate the impact of the PTM backbone on AMPA cross-reactivity detection, we compared PTM-FCS and cyclic modified peptide (CXP2/4) assays. We aim to aid the design of future studies regarding AMPAs and cross-reactivity.

## Methods

### AMPA detection in serum samples

Baseline serum samples from 608 RA patients included in the Leiden Early Arthritis Clinic (EAC) were assessed for ACPA, anti-CarP and AAPA levels as described previously [7, 15, 17, 18]. All recent-onset arthritis (less than 2 years) individuals received their diagnosis within 1 year of follow-up. Briefly, heat-inactivated FCS was modified with either chemical or enzymatic reactions to contain citrulline (Cit), homocitrulline (Hcit) or acetyllysine (Ac). Citrullination was performed as previously described with minor alterations [15]. Protein (1 mg/mL) was incubated overnight at 37 °C in 20 mM Tris–HCl, 1 mM EDTA, 10 mM DTT, and 5 mM CaCl_2_. Carbamylation was induced by incubating protein (5 mg/mL) with 1 M KOCN (in PBS) for 12 h at 37 °C. Protein was subsequently desalted with Zeba spin desalting columns (Thermofisher) according to the manufacturer’s protocol. Acetylation was carried out as previously described [19]. Briefly, 20 mL of protein (1 mg/mL in Na_2_CO_3_) was incubated with 100 µL acetic anhydride and 400 µL pyridine, both added dropwise. The solution was incubated for 18 h at 30 °C under gentle agitation. To stop the reaction, 400 µL of 1 M Tris was added per 20 mL of solution. Proteins were concentrated using concentrator columns (Pierce, 10 K MWCO) and further purified with desalting columns (Zeba, 7 K MWCO, Thermofisher). Protein modifications were validated by ELISAs as described below using monoclonal antibodies specific to one or more of the introduced modifications.

Next, Nunc maxisorb plates (Thermofisher) were coated with modified or unmodified FCS and incubated with samples overnight. Bound immunoglobulin G (IgG) was detected using rabbit anti-human IgG (Dako) and HRP-labeled goat anti-rabbit IgG (Dako). HRP enzyme activity was visualised using ABTS read at 415 nm. For peptide ELISAs, plates coated overnight with streptavidin (Invitrogen) were incubated with biotinylated modified (Cit, Hcit, Ac) or unmodified (Arg, Lys) CXP2 (Patent EP2071335) synthesised at the LUMC facility (Table S1). Following sample incubation, bound antibodies were detected with HRP-labeled goat anti-human IgG (Dako) and visualised with ABTS. Using a standard from pooled RA sera, absorbance values were interpolated into arbitrary units per milliliter (au/mL). The value of the unmodified backbone, defined in this study as the molecular scaffold (either peptide or protein) onto which the PTM was introduced, was subtracted from the value of the corresponding PTM-modified backbone to obtain the specific AMPA signal.

### Cut-off calculation and data analysis

To determine a positive response towards a specific PTM backbone, 80 healthy donor samples were measured to calculate a cut-off of average au/mL plus two times the standard deviation of the specific signal (Fig S1). Samples were assigned a positive or negative status for each autoantibody and patients who tested negative on every modification across all backbones were excluded from data analysis. Data from 389 patients were analysed using Rstudio. Correlation analyses on AMPA levels measured on either backbone were performed in GraphPad Prism.

### Statistical analysis

The 389 EAC patients were divided into double positive, single positive, or negative groups depending on their PTM reactivity on each backbone. A McNemar’s test was used to calculate whether patient PTM reactivity differed significantly per backbone used. All analyses were performed in Rstudio.

### AMPA isolation

AMPAs were isolated from sera of three RA patients and one healthy donor as a control. Nunc maxisorb plates (Thermofisher) were coated with FCS containing either Cit, Hcit or Ac modifications, and incubated with samples (24 µL serum) overnight. For the peptide-based capturing, wells of a streptavidin coated plate (Microcoat) were incubated with biotinylated modified (Cit, Hcit, Ac) CXP2 (Patent EP2071335) or CXP4 synthesised at the LUMC peptide synthesis facility (Table S1). Samples were eluted with 0.1 M acetic acid, followed by neutralisation with 1 M Tris–HCl (pH 9). The samples were then loaded onto Fc-specific beads (CaptureSelect FcXL, Thermofisher) and incubated overnight, followed by the same elution and neutralisation process. Eluted samples were titrated and measured for AMPA reactivity on FCS- or CXP2/4-based ELISAs. AMPA reactivity profiles were generated by normalising ACPA, anti-CarP, and AAPA reactivities per sample, reflecting the epitope recognition profile. This was achieved by combining the area under the curves (AUC) from the titration or absorbances for each of the reactivities and expressing them as a proportion of the total reactivity.

## Results

### Detection of multiple AMPA reactivities is increased with CXP2 compared to PTM-FCS

To evaluate the impact of PTM backbones on AMPA detection, we measured three autoantibody reactivities in sera of 389 EAC patients using both protein- (FCS) and peptide- (CXP2) based assays. Patients were stratified as positive or negative for each individual assay and then grouped by backbone to be classified as single-positive, double-positive, or negative.

**Fig. 1:**
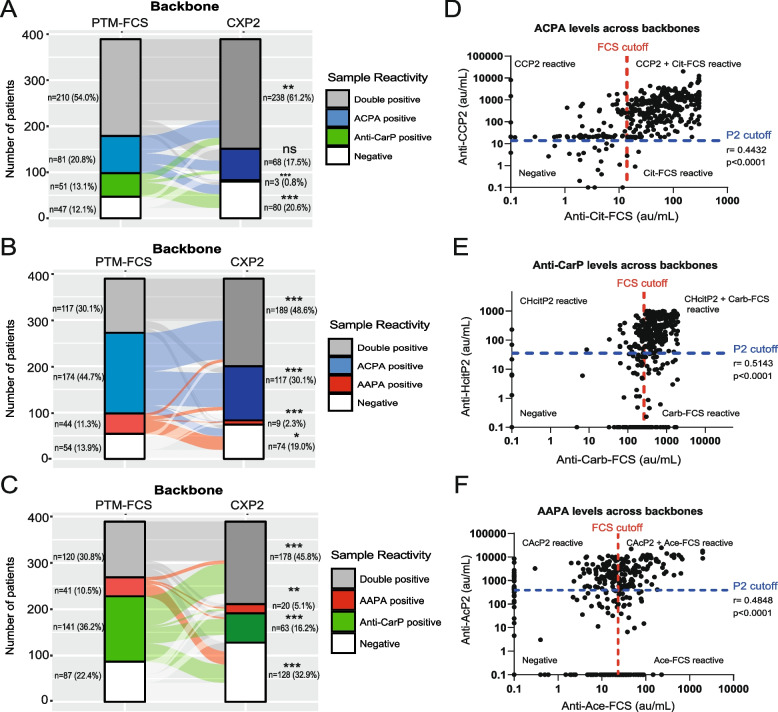
Detection of AMPA reactivity differs between antigen backbones. Sera from 389 patients were analysed for presence of ACPAs, anti-CarPs, and AAPAs using protein (FCS) or peptide (CXP2)-based backbones. Patients were stratified as positive or negative for each autoantibody. Sankey diagrams depict changes in reactivities towards (**A**) Citrullination vs Carbamylation, (**B**) Carbamylation vs Acetylation or (**C**) Citrullination vs Acetylation on the FCS or CXP2 backbone. A McNemar’s test was used to assess the significance of each reactivity (double positive, single positive, or negative groups) between backbones. Correlation between (**D**) ACPA (**E**) Anti-CarP and (**F**) AAPA levels (au/mL) is plotted for either backbone. The red line indicates the PTM-FCS cut-off and the blue line marks the CXP2 cut-off for each assay. Samples with a value of 0 au/mL were replaced with 0.1 au/mL for visualisation on the logarithmic scale. ns=not significant, *p ≤0.05, **p ≤ 0.01, ***p ≤ 0.001. PTM, post-translational modification; FCS, fetal calf serum; CXP2, cyclic modified peptide 2; ACPA, anti-citrullinated protein antibodies; AAPA, anti-acetylated protein antibodies; Anti-CarP, anti-carbamylated protein antibodies; CCP2, cyclic citrullinated peptide 2; CHcitP2, cyclic homocitrullinated peptide 2; CAcP2, cyclic acetylated peptide 2; Cit-FCS, citrullinated-FCS; Carb-FCS, carbamylated-FCS; Ace-FCS, acetylated-FCS

To visualise shifts in individual patient reactivity against PTM-FCS and CXP2, we used Sankey diagrams. When comparing anti-citrullinated versus anti-carbamylated backbone reactivities, nearly half of the single positive ACPA patients on FCS-based backbones shifted to the double-positive category on CXP2 (Fig. 1A). Significantly more patients were found to be anti-CarP single positive on PTM-FCS assays (13.1%) compared to CXP2 (0.8%) (Fig. 1A). When comparing anti-citrullinated versus anti-acetylated backbone reactivity, double-positive patients also increased from 30.1% on PTM-FCS to 48.6% on CXP2 (Fig. 1B). Similarly, in anti-carbamylated versus anti-acetylated backbone reactivity, CXP2 yielded significantly higher detection of samples reactive to both modified peptides (45.8%) compared to PTM-FCS (30.8%) (Fig. 1C). The same trend persisted when comparing all three backbone reactivities for each assay with triple positive patients increasing from 26.0% on PTM-FCS to 45% on CXP2 (Fig. S2).

Despite the increased multi-reactivity on CXP2, the proportion of non-reactive samples was also consistently higher compared to PTM-FCS, highlighting different detection sensitivities between the two assays (Fig. 1A-C). To determine whether the apparent discrepancy observed between the assays might be due to imprecise measurements surrounding the cut-off, we compared AMPA levels across both backbones. While AMPA levels based upon the two assays correlated moderately in many patients, some showed clear backbone-based level differences (Fig. 1D-F). Thus, apparent discrepant findings between the assays did not appear to be largely attributable to borderline values around the cut-off limits. Rather, our results suggest that PTM backbones can significantly influence AMPA reactivity profiles, with peptide-based assays increasing detection of reactivities towards multiple PTM residues.

### Modified FCS captures AMPAs with a more restricted epitope recognition profile compared to CXP2

**Fig. 2:**
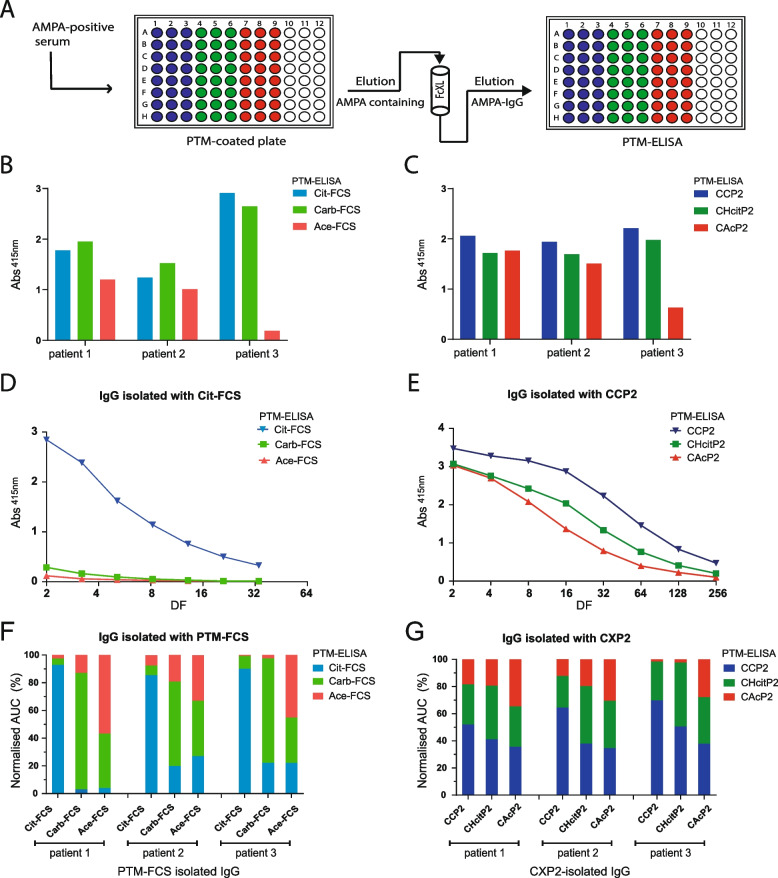
AMPA capturing and detection with different antigen backbones alters epitope specificities. **A** AMPAs were isolated from patient serum using either modified FCS or CXP2, followed by IgG capturing. Epitope recognition profiles were assessed using a PTM-ELISA. Reactivity to PTMs was detected in the starting material (serum was diluted 50 times) by (**B**) FCS and (**C**) peptide-based ELISAs. **D** Titration of IgG sample isolated with Cit-FCS and detected by modified FCS ELISA (patient 1). **E** Titration of IgG sample isolated with CCP2 and detected by CXP2 ELISA (patient 1). **F** AMPA reactivity profiles per patient for IgG isolated with either Cit-FCS, Carb-FCS, or Ace-FCS. The samples were detected by a modified FCS ELISA. **G** AMPA reactivity profiles per patient for IgG isolated with either CCP2, CHcitP2, or CAcP2. The samples were detected by CXP2 ELISA. IgG, immunoglobulin G; AMPA, anti-modified protein antibodies; PTM-ELISA, post-translational modification enzyme-linked immunosorbent assay; CXP2, cyclic modified peptide 2; CCP2, cyclic citrullinated peptide 2; CHcitP2, cyclic homocitrullinated peptide 2; CAcP2, cyclic acetylated peptide 2; FCS, fetal calf serum; Cit-FCS, citrullinated-FCS; Carb-FCS, carbamylated-FCS; Ace-FCS, acetylated-FCS

Next, we aimed to investigate whether the enhanced detection of multiple AMPA reactivities with CPX2-based ELISAs compared to FCS-based assays can be attributed to increased detection of cross-reactive AMPA. To this end, we selected three patients with high AMPA values, isolated AMPAs from serum using either modified FCS or CXP2, followed by IgG purification, and assessed the reactivity of the isolated antibodies towards the three different PTMs (Fig. 2A). The starting material contained reactivity to all three PTMs, as determined by both FCS- and peptide-based ELISAs (Fig. 2B and C, respectively). Following AMPA-IgG isolation, samples were titrated and reactivity was reassessed (Fig. 2D and E).

AMPA reactivity profiles were generated to provide a comparative overview of antibody responses against different post-translational modifications (Fig. 2F and G). First, ACPA, anti-CarP, and AAPA reactivities from each sample were quantified by calculating the AUC values from titration assays. These values reflect the strength of each antibody response. The AUCs for ACPA, anti-CarP, and AAPA were then summed to obtain the total AMPA reactivity value for each sample. Finally, each individual AUC was expressed as a proportion of this total, yielding a normalised reactivity profile that illustrates the relative contribution of each antibody specificity (ACPA, anti-CarP, and AAPA) within the overall AMPA response. No AMPA reactivity was detected against unmodified CXP2 (arginine or lysine), and healthy donor serum displayed no reactivity in the assays (Fig. S3). ACPA-IgG isolated from patient 1 with Cit-FCS predominantly exhibited anti-Cit-FCS reactivity (Fig. 2D), whereas ACPA-IgG isolated from the same patient with CCP2 showed a more cross-reactive epitope recognition profile (Fig. 2E). Similar findings were observed across all three patients, where isolation with modified FCS led to enrichment of antibodies against the specific PTM used for isolation, while CXP2-isolated antibodies broadly recognised different PTMs (Fig. 2F and G, respectively). Individual patient PTM-reactivity profiles were confirmed to be highly consistent when using either CXP2 or CXP4 for isolation and detection (Fig. 2G and Fig. S4), indicating that reactivity is maintained across both peptide backbones. Since CXP4 has a defined sequence, it allows assessment of PTM reactivity in a clear sequence context. Validating our findings against the widely used but sequence-undefined CXP2 confirms that the observed reactivity patterns are not limited to a single synthetic peptide. Overall, these findings show that the antigen backbone influences AMPA isolation as modified FCS captures AMPAs with a more restricted, less cross-reactive epitope recognition profile compared to those captured by CXP2/4.

**Fig. 3:**
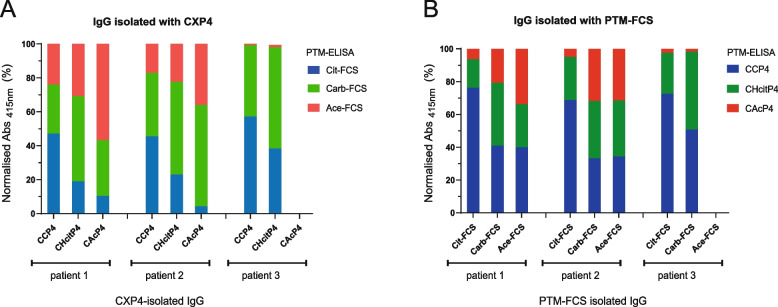
Antigen backbone influences both the isolation and the detection of AMPA cross-reactivity. **A** AMPA reactivity profiles per patient for IgG isolated with either CCP4, CHcitP4, or CAcP4. The samples were detected by a modified FCS ELISA. **B** AMPA reactivity profiles per patient for IgG isolated with either Cit-FCS, Carb-FCS, or Ace-FCS. The samples were detected by a CXP4 ELISA. Samples isolated with CAcP4 and Ace-FCS from patient 3 were excluded due to values being too low. IgG, immunoglobulin G; AMPA, anti-modified protein antibodies; PTM-ELISA, post-translational modification enzyme-linked immunosorbent assay; CXP4, cyclic modified peptide 4; CCP4, cyclic citrullinated peptide 4; CHcitP4, cyclic homocitrullinated peptide 4; CAcP4, cyclic acetylated peptide 4; FCS, fetal calf serum; Cit-FCS, citrullinated-FCS; Carb-FCS, carbamylated-FCS; Ace-FCS, acetylated-FCS

To evaluate whether this difference was due to the detection or isolation procedure, we next conducted a “backbone-switch ELISA”. In these experiments, AMPAs were isolated with one backbone (e.g. FCS) followed by detection in an ELISA with another antigen backbone (e.g. CXP4) (Fig. 3). AMPA-IgG isolated with a peptide (CXP4) and detected by FCS-based ELISA displayed a diverse recognition profile (Fig. 3A). This shows that the CXP4-isolated AMPAs remained highly cross-reactive with various PTMs, even when detected using a protein-based ELISA. Isolation of AMPA-IgG with PTM-FCS and detection by peptide-based ELISA (Fig. 3B) appeared to increase the diversity of the recognition profile compared to that obtained with FCS-based ELISA (Fig. 2F). Overall, these findings emphasise that the antigen backbone influences both the isolation of AMPA and the detection of AMPA cross-reactivity.

## Discussion

This study shows that differences in the PTM backbone used in AMPA detection assays significantly influence observed serum reactivity profiles, highlighting how structural variations shape the detection and characterisation of AMPA-responses. CXP2-based assays identify a higher proportion of patients with serum reactivity to multiple PTM residues, whereas FCS-based assays detect a more restricted AMPA profile. The antigen backbone also affects AMPA isolation, with modified FCS capturing AMPAs that exhibit a more restricted, less cross-reactive epitope recognition profile compared to antibodies isolated with modified CXP2. These findings suggest that different antigen backbones can selectively enrich for subsets of AMPAs with distinct fine specificity and cross-reactivity patterns.

Consistent with our findings, previous studies have demonstrated that RA patient sera contain both cross-reactive AMPAs and those with distinct specificities [15, 16]. Specifically, autoantibodies recognising carbamylated, but not citrullinated, protein antigens have been identified [18, 20]. Interestingly, a previous study showed that monoclonal AMPAs exhibit higher cross-reactivity in CXP-based ELISAs compared to PTM-fibrinogen-based ELISAs [16]. This aligns with our finding of decreased AMPA cross-reactivity observed in polyclonal antibodies isolated from sera with PTM-FCS and suggests that this phenomenon likely also extends to other defined protein backbones.

Research into the structural basis of ACPA-reactivity indicates that residues flanking the citrulline-moiety can have an impact on binding patterns [21, 22]. Factors like motifs, such as Cit–Gly, enhance the likelihood of a favourable binding interaction [13, 23]. However, the proximity of structural elements in peptides, such as a biotin tag located near the citrulline residue, can also have a large influence on ACPA detection assay performance [23]. The nature and magnitude of the secondary contacts formed between AMPAs and additional residues likely affect the extent of cross-reactivity [23, 24]. Synthetic PTM peptides offer precise control over epitope presentation and positioning, which could enhance cross-reactivity detection, particularly if the PTMs in these peptides share the same or similar flanking residues. Indeed, in CXPs, the modified residue (e.g., citrulline or homocitrulline) is introduced at the exact same position within an identical sequence context. In contrast, in protein-rich sources like FCS, arginine and lysine residues are likely distributed in distinct regions of the protein. As a result, citrullination, acetylation, and carbamylation may occur at different positions, leading to non-overlapping epitope contexts with variability in the flanking PTM residues, potentially limiting cross-reactivity detection. Additionally, peptides are often cyclised, a modification that has been shown to increase assay sensitivity, underscoring the importance of an optimal conformation for enhanced AMPA detection [25]. The optimised nature of peptide-based assays enables maximum detection of RA patients, and likely facilitates more permissive epitope recognition by AMPAs. In this context, the spacing, abundance, and location of modifications are likely crucial factors influencing reactivity—aspects that are unknown for PTM-FCS.

Interestingly, in some patients, AMPA reactivity towards a specific PTM residue (e.g. carbamylation) was only detected in the FCS-based assay and not in the CXP2-based assay, suggesting that the antibodies reacting in either assay may have distinct fine specificities (Fig. 1A, E). This aligns with a study showing that ACPA monoclonal antibodies can exhibit diverse fine specificities, with many recognising peptides sharing similar features like a Cit–Gly site, while subsets display restricted binding to peptides from Cit-vimentin, which lack these features [13]. Consistent with this finding, it was previously shown that some ACPAs are “promiscuous,” cross-reacting with various citrullinated peptides, while others are “private,” likely requiring specific amino acid side chains for protein-specific interactions [26]. Our data support this spectrum of reactivity, showing that AMPA reactivity profiles vary depending on the antigen backbone. The observed differences in AMPA reactivities between FCS- and CXP2-based assays suggest variations in fine specificity, with some antibodies exhibiting broader (promiscuous) recognition and others requiring more specific interactions (private). Potentially, CXPs preferentially capture antibodies that rely on common or minimal sequence features, whereas FCS may favour private antibodies requiring additional, more specific side-chain interactions. Future studies could further characterise the spectrum of AMPA reactivities and investigate the functional roles of both private and promiscuous AMPAs in relation to pathogenicity [26].

A limitation of our study is the unknown antigen density and structure of PTM-FCS. The unknown antigen composition within this protein-rich backbone complicates comparison with modified peptides and hinders understanding of how it affects cross-reactivity detection. Furthermore, the heterogeneous protein content of FCS may inherently contain modified or immunogenic proteins, potentially contributing to varying levels of non-specific binding. To minimise this background effect, our study included non-modified FCS alongside the PTM-FCS. Reactivity to the non-modified FCS was subtracted from that of the PTM-FCS, providing a reliable approach to account for background signal and ensure that the measured responses reflect PTM-specific antibody reactivity. Additionally, our study utilises a large patient cohort to demonstrate the impact of PTM backbones on AMPA reactivity, with findings validated through AMPA purification assays that underscore the broad influence of PTM backbones across assays.

Our study shows that the PTM backbone significantly influences AMPA reactivity profiles, with distinct antibody reactivity patterns detected depending on the antigen backbone used. This variability underscores the necessity of considering the specific properties of each PTM backbone in autoantibody research as they can substantially affect experimental outcomes and interpretations. From a clinical perspective, these findings highlight the importance of backbone selection in assay development, which may influence both diagnostic performance and patient stratification. Ultimately, the (cross-)reactivity patterns of AMPAs could offer valuable insights into the evolution of autoimmune responses and may help elucidate the mechanisms driving the breakdown of tolerance to modified antigens in autoimmune diseases.

## Supplementary Information

Supplementary Material 1. Figure S1: AMPA levels in healthy controls. AMPA levels (ACPA, anti-CarP, and AAPA) were measured in 80 healthy controls using either PTM-FCS- or CXP2-based ELISAs. Results are reported in arbitrary units per millilitre (au/mL), with group means indicated. Positivity cut-offs for each assay were defined as the mean au/mL plus two times the standard deviation of the healthy control group. PTM, post-translationally modified; FCS, fetal calf serum; CXP2, cyclic modified peptide 2; ACPA, anti-citrullinated protein antibody; Anti-CarP, anti-carbamylated protein antibody; AAPA, anti-acetylated protein antibody. Figure S2: Differential AMPA detection across distinct PTM backbones. Sera from 389 patients were analysed for the presence of ACPAs, anti-CarPs, and AAPAs using protein (FCS) or peptide (CXP2)-based backbones. Patients were stratified as positive or negative for each autoantibody. Sankey diagrams depict changes in reactivities towards all three PTMs on the FCS or CXP2 backbone. PTM, post-translational modification; FCS, fetal calf serum; CXP2, cyclic modified peptide 2; ACPA, anti-citrullinated protein antibodies; AAPA, anti-acetylated protein antibodies; Anti-CarP, anti-carbamylated protein antibodies. Figure S3: AMPA capturing controls. CXP2 IgG ELISA of A) CCP2-, B) CHcitP2- and C) CAcP2-isolated IgG samples from serum of three patients and one healthy donor. Captured antibody samples were diluted eight times. HD, healthy donor; IgG, immunoglobulin G; CXP2, cyclic modified peptide 2; CCP2, cyclic citrullinated peptide 2; CHcitP2, cyclic homocitrullinated peptide 2; CAcP2, cyclic acetylated peptide 2; CArgP2, cyclic arginine peptide 2 (unmodified); CLysP2, cyclic lysine peptide 2 (unmodified). Figure S4: Detection of AMPA reactivity with CXP4. AMPAs were isolated from patient serum using modified CXP4, followed by IgG capturing. For each patient, AMPA reactivity profiles were determined for IgG isolated using CCP4, CHcitP4, or CAcP4. Detection was performed using a CXP4-based ELISA. IgG, immunoglobulin G; AMPA, anti-modified protein antibodies; PTM-ELISA, post-translational modification enzyme-linked immunosorbent assay; CXP4, cyclic modified peptide 4; CCP4, cyclic citrullinated peptide 4; CHcitP4, cyclic homocitrullinated peptide 4; CAcP4, cyclic acetylated peptide 4.

Supplementary Material 2

## Data Availability

No datasets were generated or analysed during the current study.

## Abbreviations

AAPA: Anti-acetylated protein antibody
Ac: Acetyllysine
ACPA: Anti-citrullinated protein antibody
AMPA: Anti-modified protein antibody
Anti-CarP: Anti-carbamylated protein antibody
Arg: Arginine
AUC: Area under curve
Cit: Citrulline
CXP2/4: Cyclic modified peptide second/fourth generation
EAC: Early arthritis clinic
ELISA: Enzyme-linked immunosorbent assay
FCS: Fetal calf serum
Hcit: Homocitrulline
Lys: Lysine
PTM: Post-translational modification
RA: Rheumatoid arthritis
