# Supplementary figures and images for: Insights into the detection of AMPA cross-reactivity: comparing cyclic peptide- to protein-based assays
Source: Arthritis Res Ther. 2025 Jul 7;27:138. doi: 10.1186/s13075-025-03591-y (PMC12232869; doi:10.1186/s13075-025-03591-y)

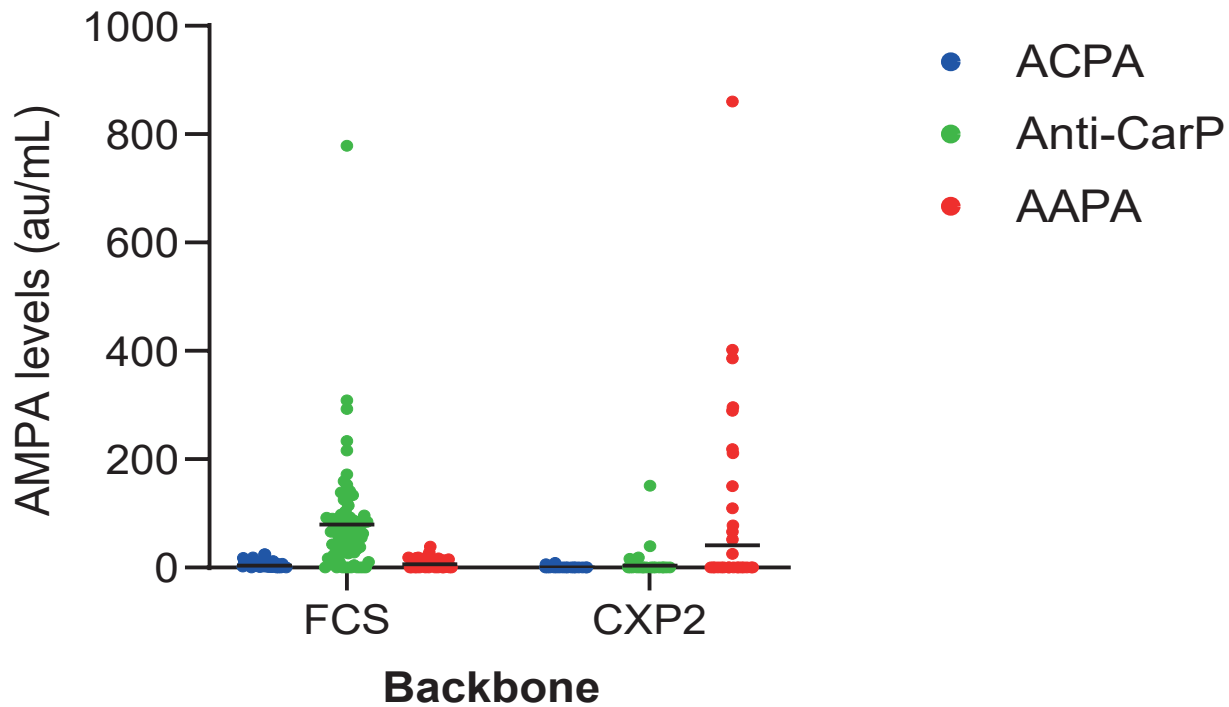

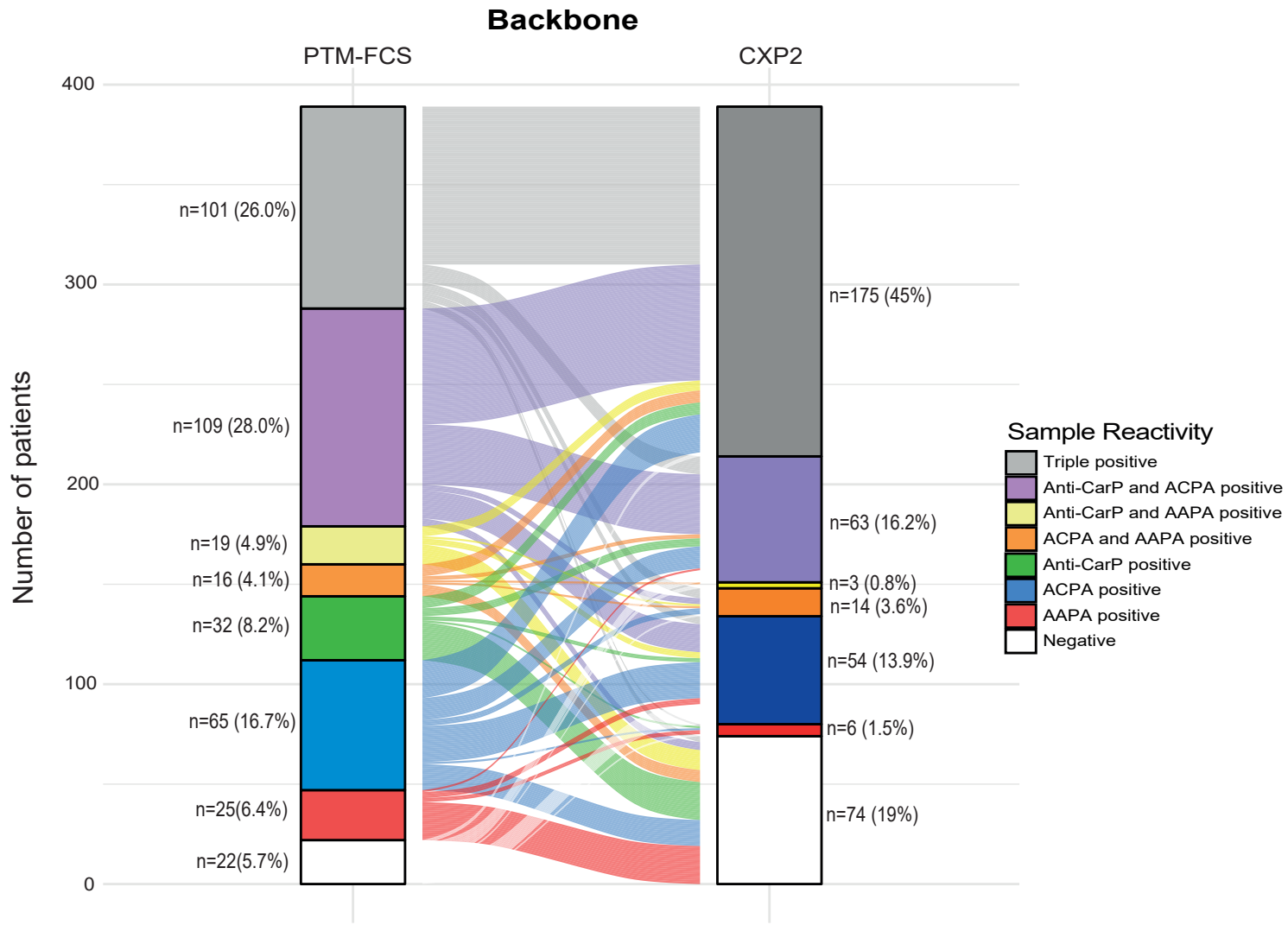

**A****Captured anti-CCP2 IgG**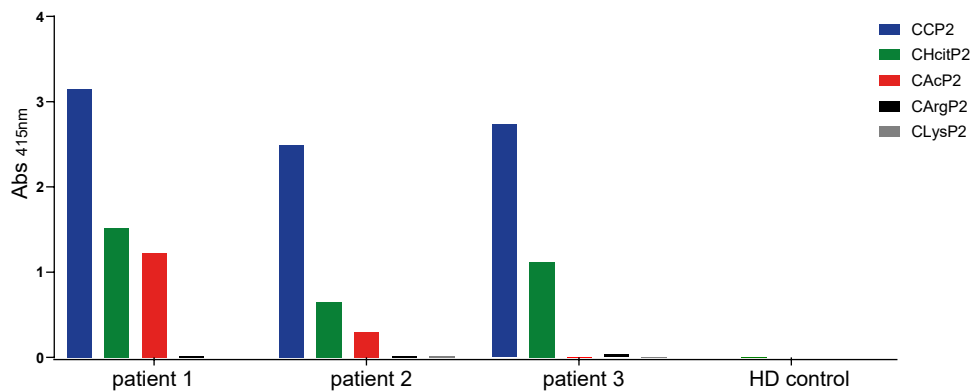**B****Captured anti-CHcitP2 IgG**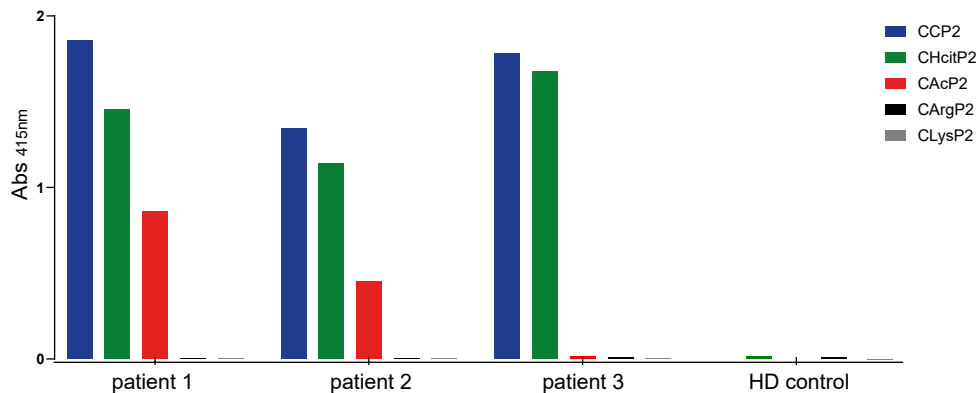**C****Captured anti-CAcP2 IgG**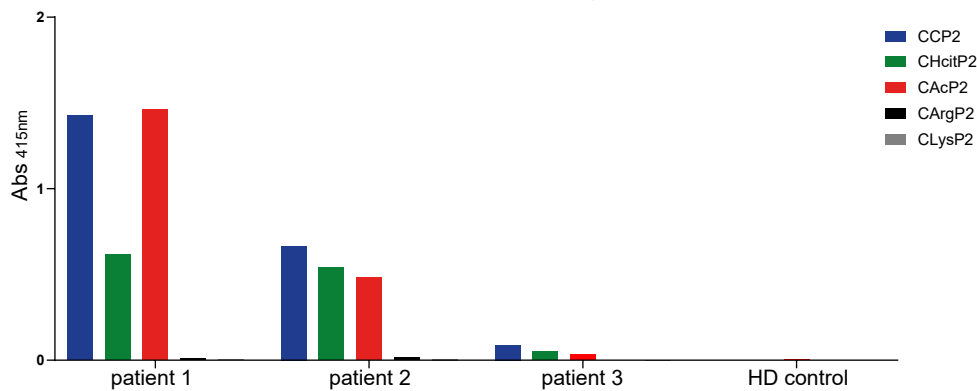

## IgG isolated with CXP4

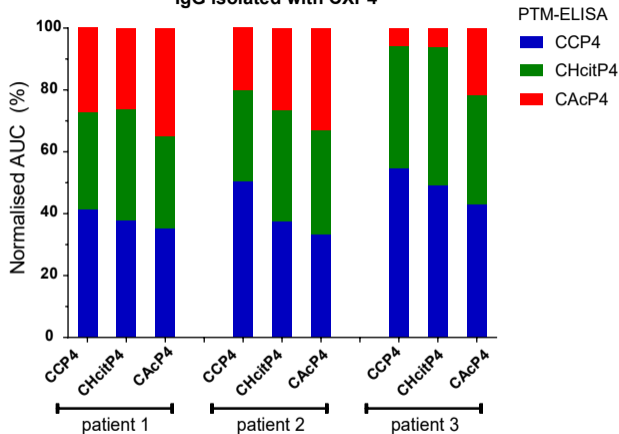

Supplement: Supplementary file 1 — Supplementary Material 1. Figure S1: AMPA levels in healthy controls. AMPA levels (ACPA, anti-CarP, and AAPA) were measured in 80 healthy controls using either PTM-FCS- or CXP2-based ELISAs. Results are reported in arbitrary units per millilitre (au/mL), with group means indicated. Positivity cut-offs for each assay were defined as the mean au/mL plus two times the standard deviation of the healthy control group. PTM, post-translationally modified; FCS, fetal calf serum; CXP2, cyclic modified peptide 2; ACPA, anti-citrullinated protein antibody; Anti-CarP, anti-carbamylated protein antibody; AAPA, anti-acetylated protein antibody. Figure S2: Differential AMPA detection across distinct PTM backbones. Sera from 389 patients were analysed for the presence of ACPAs, anti-CarPs, and AAPAs using protein (FCS) or peptide (CXP2)-based backbones. Patients were stratified as positive or negative for each autoantibody. Sankey diagrams depict changes in reactivities towards all three PTMs on the FCS or CXP2 backbone. PTM, post-translational modification; FCS, fetal calf serum; CXP2, cyclic modified peptide 2; ACPA, anti-citrullinated protein antibodies; AAPA, anti-acetylated protein antibodies; Anti-CarP, anti-carbamylated protein antibodies. Figure S3: AMPA capturing controls. CXP2 IgG ELISA of A) CCP2-, B) CHcitP2- and C) CAcP2-isolated IgG samples from serum of three patients and one healthy donor. Captured antibody samples were diluted eight times. HD, healthy donor; IgG, immunoglobulin G; CXP2, cyclic modified peptide 2; CCP2, cyclic citrullinated peptide 2; CHcitP2, cyclic homocitrullinated peptide 2; CAcP2, cyclic acetylated peptide 2; CArgP2, cyclic arginine peptide 2 (unmodified); CLysP2, cyclic lysine peptide 2 (unmodified). Figure S4: Detection of AMPA reactivity with CXP4. AMPAs were isolated from patient serum using modified CXP4, followed by IgG capturing. For each patient, AMPA reactivity profiles were determined for IgG isolated using CCP4, CHcitP4, or CAcP4. De [file 13075_2025_3591_MOESM1_ESM.pdf]
