# Supplementary material for: Insights into the detection of AMPA cross-reactivity: comparing cyclic peptide- to protein-based assays
Source: Arthritis Res Ther. 2025 Jul 7;27:138. doi: 10.1186/s13075-025-03591-y (PMC12232869; doi:10.1186/s13075-025-03591-y)
Supplement: Supplementary file 2 — Supplementary Material 2 [file 13075_2025_3591_MOESM2_ESM.docx]

**Table S1: Peptide sequences**

| Peptide | Sequence |
| --- | --- |
| CCP2, CArgP2, CHCitP2, CLysP2 CAcP2 (cyclic) | Patent EP2071335 |
| CCP4, CArgP4, CHcitP4, CLysP2, CAcP4 (cyclic) | HQFRF**X**GNleSRAACZO |

X = arginine, lysine, citrulline, homocitrulline, acetyllysine

Z = 6-aminohexanoic acid

O = lys (biotin)-amide

Nle= norleucine

Ttds-linker = 1,13-diamino-4,7,10-trioxatridecane succinimic acid linker

CCP2: anti-cyclic citrullinated peptide 2, CArgP2 anti-cyclic arginine peptide 2, CHcitP2: anti-cyclic homocitrullinated peptide 2, CArgP2 anti-cyclic lysine peptide 2, CAcP2: anti-cyclic acetylated peptide 2

CCP4: anti-cyclic citrullinated peptide 4, CArgP4 anti-cyclic arginine peptide 4, CHcitP4: anti-cyclic homocitrullinated peptide 4, CLysP4: anti-cyclic lysine peptide 4, CAcP4: anti-cyclic acetylated peptide 4

Cyclisation of CXP4 was achieved by coupling a chloroacetyl group to the N-terminus, this reacts with a cysteine forming a thioether bond.
